# Various Auto-Correlation Functions of *m*-Bit Random Numbers Generated from Chaotic Binary Sequences

**DOI:** 10.3390/e23101295

**Published:** 2021-09-30

**Authors:** Akio Tsuneda

**Affiliations:** Division of Informatics and Energy, Faculty of Advanced Science and Technology, Kumamoto University, Kumamoto 860-8555, Japan; tsuneda@cs.kumamoto-u.ac.jp; Tel.: +81-96-342-3853

**Keywords:** random number generation, auto-correlation function, m-bit sequence, chaotic binary sequence

## Abstract

This paper discusses the auto-correlation functions of *m*-bit random numbers obtained from *m* chaotic binary sequences generated by one-dimensional nonlinear maps. First, we provide the theoretical auto-correlation function of an *m*-bit sequence obtained by *m* binary sequences that are assumed to be uncorrelated to each other. The auto-correlation function is expressed by a simple form using the auto-correlation functions of the binary sequences. This implies that the auto-correlation properties of the *m*-bit sequences can be easily controlled by the auto-correlation functions of the original binary sequences. In numerical experiments using a computer, we generated *m*-bit random sequences using some chaotic binary sequences with prescribed auto-correlations generated by one-dimensional chaotic maps. The numerical experiments show that the numerical auto-correlation values are almost equal to the corresponding theoretical ones, and we can generate *m*-bit sequences with a variety of auto-correlation properties. Furthermore, we also show that the distributions of the generated *m*-bit sequences are uniform if all of the original binary sequences are balanced (i.e., the probability of 1 (or 0) is equal to 1/2) and independent of one another.

## 1. Introduction

Chaos-based random number generation has been widely studied [1,2,3,4,5,6,7,8,9,10,11,12,13,14]. In most cases, the random numbers are desired to be independent (or uncorrelated), which are often called *truly random numbers*, especially for security applications. However, correlated random numbers are also useful in Monte-Carlo methods [15,16] for simulating various kinds of stochastic phenomena. Using one-dimensional (1-D) chaotic maps, we can design various kinds of chaotic sequences including uncorrelated and correlated ones [1,8,9,11,16], which can be adapted according to the desired properties depending on the applications. For example, the convergence rate of Monte-Carlo integrations can be increased by using chaotic sequences with proper auto-correlations, which is called *super-efficient chaotic Monte-Carlo simulations* [16]. Thus, it is very important to control or design the statistical properties of random numbers according to each application. Noting that random numbers in such numerical methods using computers are represented by finite bits (64-bit, 32-bit, etc.), we discuss *m*-bit random numbers generated by *m* different binary random sequences in this paper. The statistical properties of the *m*-bit random numbers depend on the original *m* binary sequences. Thus, by changing the properties of the original binary sequences, we can generate *m*-bit random sequences with a variety of statistical properties. The main purpose of this paper is to discuss the controllability of the auto-correlations of the *m*-bit random numbers but not to generate only *purely random* numbers. In [14], we discussed the case of m=2, and we generalize the results of [14] for general integers m≥2 in this paper. First, we provided the theoretical auto-correlation function of the *m*-bit sequence generated by *m* binary sequences. Next, we performed some numerical experiments generating *m*-bit sequences from several types of chaotic binary sequences generated by 1-D chaotic maps. The numerical auto-correlation functions and distributions of the generated *m*-bit sequences are also shown.

The rest of this paper is organized as follows. In Section 2, we provide the method for generating an *m*-bit sequence from *m* binary sequences. The theoretical auto-correlation function of the *m*-bit sequence is also provided. In Section 3, two types of chaotic maps for generating uncorrelated/correlated chaotic binary sequences are introduced, as examples. Some numerical results are shown in Section 4. Finally, the concluding remarks are presented in Section 5.

## 2. Auto-Correlation Functions of m-Bit Random Sequences

### 2.1. Definition of Auto-Correlation Function

For a general random sequence ${\left\{Z_{n}\right\}}_{n=0}^{\infty }$, its auto-correlation function $R(\ell ;Z_{n})$ is defined by

(1) $$\begin{array}{cc} R(\ell ;Z_{n}) & =E[\left(Z_{n}-E\left[Z_{n}\right]\right)\left(Z_{n+\ell }-E\left[Z_{n}\right]\right)],\\ \; & =E\left[Z_{n}Z_{n+\ell }\right]-E{\left[Z_{n}\right]}^{2}\;\left(\ell =0,1,2,\cdots \right), \end{array}$$

where E[·] denotes taking the average and *ℓ* denotes a time delay. Additionally, the normalized auto-correlation function is defined by

(2) $$C\left(\ell ;Z_{n}\right)=\frac{R(\ell ;Z_{n})}{R(0;Z_{n})}\;\in \left[-1,1\right],$$

where $R(0;Z_{n})$ means the variance.

### 2.2. Generation of m-Bit Sequences from Binary Sequences

Let ${\left\{B_{n}^{\left(i\right)}\right\}}_{n=0}^{\infty }$(i=1,2,⋯,m) be *m* binary ({0,1}-valued) sequences. We obtain an *m*-bit random sequence ${\left\{X_{n}\right\}}_{n=0}^{\infty }$ ($\in [0,1-2^{-m}]$) by

(3) $$X_{n}=2^{-1}B_{n}^{\left(1\right)}+2^{-2}B_{n}^{\left(2\right)}+\cdots +2^{-m}B_{n}^{\left(m\right)}.$$

Now, we consider the auto-correlation function of the *m*-bit sequence provided by (3). The following theorem can be obtained.

**Theorem** **1.** *The auto-correlation function of the m-bit sequence generated by (3) is given by*

(4) $$R\left(\ell ;X_{n}\right)=4^{-1}R\left(\ell ;B_{n}^{\left(1\right)}\right)+4^{-2}R\left(\ell ;B_{n}^{\left(2\right)}\right)+\cdots +4^{-m}R\left(\ell ;B_{n}^{\left(m\right)}\right)$$

*under the assumption that any pair of the m binary sequences, ${\left\{B_{n}^{\left(i\right)}\right\}}_{n=0}^{\infty }$ and ${\left\{B_{n}^{\left(j\right)}\right\}}_{n=0}^{\infty }$(i≠j), are uncorrelated to each other such that*

(5) $$E\left[B_{n}^{\left(i\right)}B_{n+\ell }^{\left(j\right)}\right]=E\left[B_{n+\ell }^{\left(i\right)}B_{n}^{\left(j\right)}\right]=E\left[B_{n}^{\left(i\right)}\right]E\left[B_{n}^{\left(j\right)}\right]\;fori\neq jand\ell =0,1,2,\cdots .$$

**Proof.**  Substituting (3) into

(6) $$\begin{array}{cc} R(\ell ;X_{n}) & =E[\left(X_{n}-E\left[X_{n}\right]\right)\left(X_{n+\ell }-E\left[X_{n}\right]\right)],\\ \; & =E\left[X_{n}X_{n+\ell }\right]-E{\left[X_{n}\right]}^{2}, \end{array}$$

and using (5), we have

(7) $$\begin{array}{cc} R(\ell ;X_{n})= & 4^{-1}E\left[B_{n}^{\left(1\right)}B_{n+\ell }^{\left(1\right)}\right]+4^{-2}E\left[B_{n}^{\left(2\right)}B_{n+\ell }^{\left(2\right)}\right]+\cdots +4^{-m}E\left[B_{n}^{\left(m\right)}B_{n+\ell }^{\left(m\right)}\right]\\ \; & -4^{-1}E{\left[B_{n}^{\left(1\right)}\right]}^{2}-4^{-2}E{\left[B_{n}^{\left(2\right)}\right]}^{2}-\cdots -4^{-m}E{\left[B_{n}^{\left(m\right)}\right]}^{2} \end{array}$$

which gives (4). □

**Remark** **1.** *If we set ${\hat{X}}_{n}=2^{m}X_{n}$, that is*

(8) $${\hat{X}}_{n}=2^{m-1}B_{n}^{\left(1\right)}+2^{m-2}B_{n}^{\left(2\right)}+\cdots +2^{0}B_{n}^{\left(m\right)},$$

*a $2^{m}$-ary integer sequence ${\left\{{\hat{X}}_{n}\right\}}_{n=0}^{\infty }$ (${\hat{X}}_{n}\in \left\{0,1,\cdots ,2^{m}-1\right\}$) can be obtained. The auto-correlation function $R(\ell ;{\hat{X}}_{n})$ is given by*

(9) $$R\left(\ell ;{\hat{X}}_{n}\right)=4^{m}R\left(\ell ;X_{n}\right).$$

*Obviously, $C\left(\ell ;{\hat{X}}_{n}\right)=C\left(\ell ;X_{n}\right)$. In [14], the case of m=2, that is, 4-ary (quaternary) sequences, is discussed.*

**Remark** **2.** *Assume*

(10) $$E\left[B_{n}^{\left(i\right)}\right]=\frac{1}{2}\;\left(i=1,2,\cdots ,m\right),$$

*which gives*

(11) $$R\left(0;B_{n}^{\left(i\right)}\right)=\frac{1}{4}\;\left(i=1,2,\cdots ,m\right);$$

*then, we have*

(12) $$\begin{array}{cc} E[X_{n}] & =\frac{1}{2}\left(1-2^{-m}\right), \end{array}$$

(13) $$\begin{array}{cc} R(0;X_{n}) & =\frac{1}{12}\left(1-4^{-m}\right). \end{array}$$

*Furthermore, assuming*

(14) $$R\left(\ell ;B_{n}^{\left(1\right)}\right)=R\left(\ell ;B_{n}^{\left(2\right)}\right)=\cdots =R\left(\ell ;B_{n}^{\left(m\right)}\right),$$

*we have*

(15) $$\begin{array}{cc} R(\ell ;X_{n}) & =\frac{1}{3}\left(1-4^{-m}\right)R\left(\ell ;B_{n}^{\left(1\right)}\right), \end{array}$$

(16) $$\begin{array}{cc} C(\ell ;X_{n}) & =C(\ell ;B_{n}^{\left(1\right)}). \end{array}$$

**Remark** **3.** *Consider the probability that $X_{n}$ (or ${\hat{X}}_{n}$) takes a value $s_{p}$, denoted by $P(X_{n}=s_{p})$, where $s_{p}$ is a value in $2^{m}$ possible values expressed by m-bit numbers ($p=1,2,\cdots ,2^{m}$). In addition to (10), assuming that ${\left\{B_{n}^{\left(i\right)}\right\}}_{n=0}^{\infty }$(i=1,2,⋯,m) are independent of one another, we have*

(17) $$P\left(X_{n}=s_{p}\right)=\frac{1}{2^{m}}\;forallp=1,2,\cdots ,2^{m}$$

*which implies ${\left\{X_{n}\right\}}_{n=0}^{\infty }$ (or ${\left\{{\hat{X}}_{n}\right\}}_{n=0}^{\infty }$) is a sequence of random numbers with a uniform distribution.*

## 3. Chaotic Binary Sequences with Prescribed Auto-Correlations

A one-dimensional nonlinear difference equation defined by

(18) $$x_{n+1}=\tau \left(x_{n}\right),\;\;\;x_{n}\in I=\left[0,1\right],\;\;\;n=0,1,2,\cdots$$

can generate a *chaotic* real-valued sequence ${\left\{x_{n}\right\}}_{n=0}^{\infty }$ for a chaotic map τ(·) [17,18]. Additionally, we can obtain a binary sequence ${\left\{B\left(x_{n}\right)\right\}}_{n=0}^{\infty }$ using a binary function B(x)(∈{0,1}) from a real-valued sequence ${\left\{x_{n}\right\}}_{n=0}^{\infty }$. Then, the theoretical auto-correlation function of the binary sequence ${\left\{B\left(x_{n}\right)\right\}}_{n=0}^{\infty }$ is defined by

(19) $$\langle R(\ell ;B)\rangle =\int_{I}\left(B\left(x\right)-\langle B\rangle \right)\left(B\left(\tau^{\ell }\left(x\right)\right)-\langle B\rangle \right)f^{*}\left(x\right)dx,$$

under the assumption that τ(x) has an invariant density function $f^{*}\left(x\right)$, where $\tau^{\ell }\left(x\right)$ is the *ℓ*-th iterate of the map τ starting from an initial value $x=x_{0}$ and 〈B〉 denotes the average of the binary sequence ${\left\{B\left(x_{n}\right)\right\}}_{n=0}^{\infty }$ defined by

(20) $$\langle B\rangle =\int_{I}B\left(x\right)f^{*}\left(x\right)dx.$$

We also define the normalized auto-correlation function by

(21) $$\langle C\left(\ell ;B\right)\rangle =\frac{\langle R(\ell ;B)\rangle }{\langle R(0;B)\rangle }.$$

Note that, by setting $Z_{n}=B\left(x_{n}\right)$, we have the following relations:

(22) $$\begin{array}{cc} E[Z_{n}] & =\langle B\rangle , \end{array}$$

(23) $$\begin{array}{cc} R(\ell ;Z_{n}) & =\langle R(\ell ;B)\rangle , \end{array}$$

(24) $$\begin{array}{cc} C(\ell ;Z_{n}) & =\langle C(\ell ;B)\rangle . \end{array}$$

In this paper, we introduce the following two chaotic maps with I=[0,1] and $f^{*}\left(x\right)=1$, which will be used in numerical experiments later. Note that these chaotic maps are introduced just as examples and other chaotic maps can also be used.

**Figure 1:**
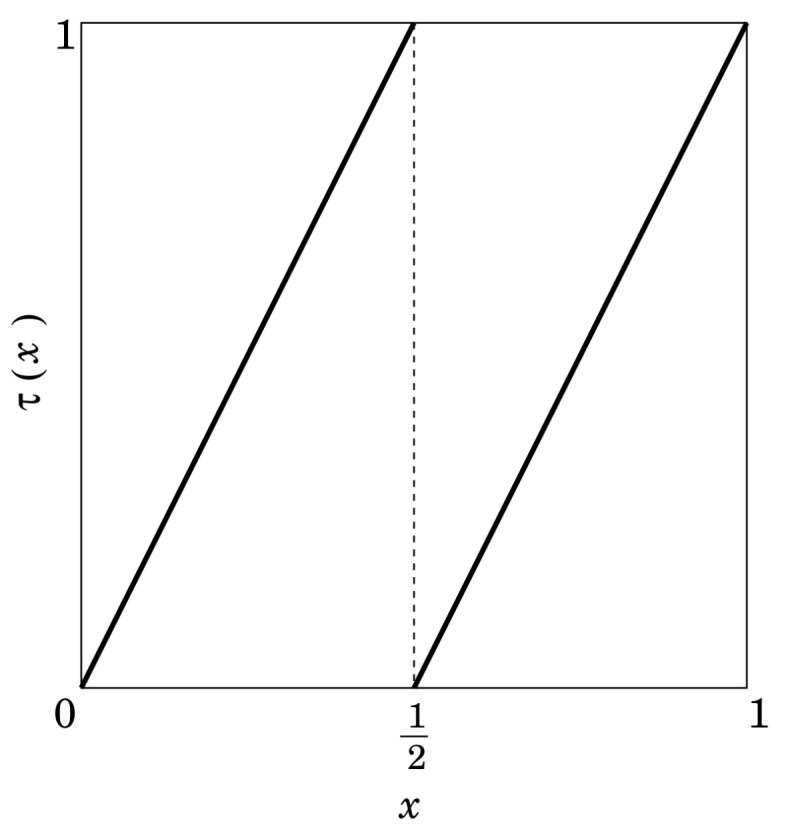
Bernoulli map.

**[Chaotic map 1]** The Bernoulli map with I=[0,1] is defined by [17,18]

(25) $$\tau \left(x\right)=\left\{\begin{array}{cc} 2x, & \left(0\leq x<\frac{1}{2}\right)\\ 2x-1 & (\frac{1}{2}\leq x\leq 1), \end{array}\right.$$

which is illustrated in Figure 1. This map has the uniform invariant density $f^{*}\left(x\right)=1$ [17].

**Figure 2:**
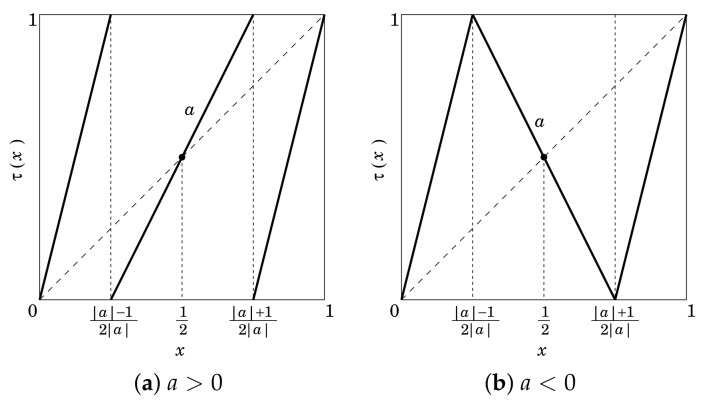
Fully stretching piecewise linear maps with 3 sections.

**[Chaotic map 2]** Define a fully stretching piecewise linear (FSPL) map with I=[0,1] as [8]

(26) $$\tau \left(x\right)=\left\{\begin{array}{cc} \frac{2|a|}{|a|-1}x & \left(0\leq x<\frac{|a|-1}{2|a|}\right)\\ ax-\frac{a-1}{2} & \left(\frac{|a|-1}{2|a|}\leq x<\frac{|a|+1}{2|a|}\right)\\ \frac{2|a|}{|a|-1}\left(x-\frac{|a|+1}{2|a|}\right) & \left(\frac{|a|+1}{2|a|}\leq x\leq 1\right), \end{array}\right.$$

where *a* is a parameter satisfying |a|>1. Examples of the map are shown in Figure 2. The FSPL maps also have the uniform invariant density $f^{*}\left(x\right)=1$ [8,18].

**Figure 3:**
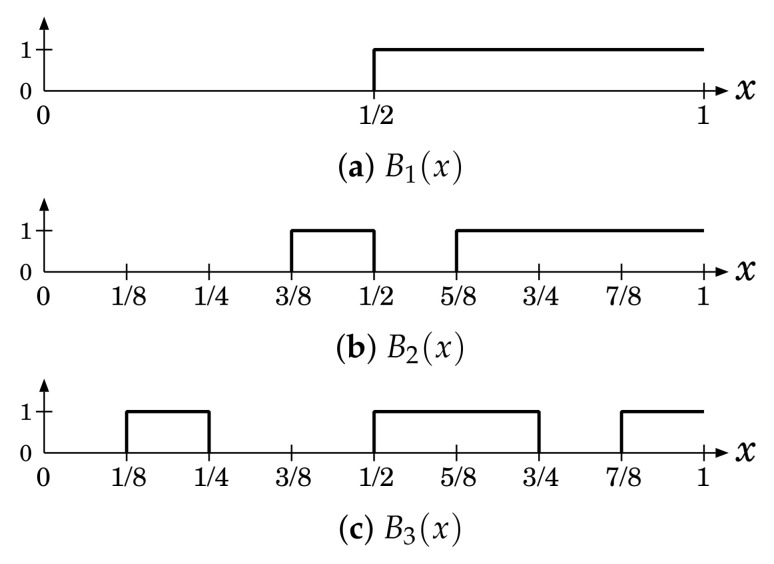
Binary functions.

In order to obtain binary sequences from chaotic real-valued sequences, we first define a *threshold function* $\Theta_{t}\left(x\right)$ as

(27) $$\Theta_{t}\left(x\right)=\left\{\begin{array}{cc} 0 & \left(x<t\right)\\ 1 & (x\geq t), \end{array}\right.$$

where *t* is a threshold. In this paper, we use the following three binary functions expressed by the threshold functions:

(28) $$\begin{array}{cc} B_{1}\left(x\right) & =\Theta_{\frac{1}{2}}\left(x\right), \end{array}$$

(29) $$\begin{array}{cc} B_{2}\left(x\right) & =\Theta_{\frac{3}{8}}\left(x\right)-\Theta_{\frac{1}{2}}\left(x\right)+\Theta_{\frac{5}{8}}\left(x\right), \end{array}$$

(30) $$\begin{array}{cc} B_{3}\left(x\right) & =\Theta_{\frac{1}{8}}\left(x\right)-\Theta_{\frac{1}{4}}\left(x\right)+\Theta_{\frac{1}{2}}\left(x\right)-\Theta_{\frac{3}{4}}\left(x\right)+\Theta_{\frac{7}{8}}\left(x\right), \end{array}$$

which are illustrated in Figure 3. Since the above two chaotic maps have $f^{*}\left(x\right)=1$, the average of the binary sequences generated by the binary functions $B_{i}\left(x\right)$ (i=1,2,3) is given by

(31) $$\langle B_{i}\rangle =\frac{1}{2}\;\left(i=1,2,3\right)$$

which gives

(32) $$\langle R\left(0;B_{i}\right)\rangle =\frac{1}{4}\;\left(i=1,2,3\right).$$

The auto-correlation function (ACF) of the generated binary sequence depends on the combination of the chaotic map and the binary function. Several examples of the auto-correlation functions are provided as follows.

**[ACF-1]** For the Bernoulli map and $B_{1}\left(x\right)$, the normalized ACF is given by [1]

(33) $$\langle C\left(\ell ;B_{1}\right)\rangle =\left\{\begin{array}{cc} 1 & \left(\ell =0\right)\\ 0 & (\ell \geq 1). \end{array}\right.$$

Note that the generated binary sequences are *i.i.d.*(independent and identically distributed).

**[ACF-2]** For the Bernoulli map and $B_{2}\left(x\right)$, the normalized ACF is given by [9,11]

(34) $$\langle C\left(\ell ;B_{2}\right)\rangle =\left\{\begin{array}{cc} 1 & (\ell =0)\;\\ \frac{1}{2} & (\ell =1)\;\\ \frac{1}{4} & (\ell =2)\;\\ 0 & (\ell \geq 3). \end{array}\right.$$

**[ACF-3]** For the Bernoulli map and $B_{3}\left(x\right)$, the normalized ACF is given by [9,11]

(35) $$\langle C\left(\ell ;B_{3}\right)\rangle =\left\{\begin{array}{cc} 1 & (\ell =0)\;\\ -\frac{1}{2} & (\ell =1)\;\\ \frac{1}{4} & (\ell =2)\;\\ 0 & (\ell \geq 3). \end{array}\right.$$

**[ACF-4]** For the FSPL map and $B_{1}\left(x\right)$, the normalized ACF is given by [8]

(36) $$\langle C\left(\ell ;B_{1}\right)\rangle =\frac{1}{a^{\ell }}.$$

By changing the value of *a*, we can control the auto-correlation property with exponential decay.

## 4. Numerical Experiments

We performed some numerical experiments using the chaotic maps and the binary functions given in the previous section. First, we generated *m* chaotic binary sequences $\left\{B\left(x_{n}^{\left(i\right)}\right)\right\}$(i=1,2,⋯,m), where $x_{0}^{\left(1\right)},x_{0}^{\left(2\right)},\cdots ,x_{0}^{\left(m\right)}$ are different initial values for the assumptions of (5) (uncorrelation) and Remark 3 (independency). Next, we obtained an *m*-bit sequence as

(37) $$X_{n}=2^{-1}B\left(x_{n}^{\left(1\right)}\right)+2^{-2}B\left(x_{n}^{\left(2\right)}\right)+\cdots +2^{-m}B\left(x_{n}^{\left(m\right)}\right).$$

We generated eight kinds of *m*-bit sequences using eight combinations of chaotic binary sequences with ACF-1, 2, 3, and 4 as in Table 1. Here, we used the numerical auto-correlation function defined by

(38) $$\hat{R}\left(\ell ;X_{n}\right)=\frac{1}{N}\sum_{n=0}^{N-1}\left(X_{n}-E\left[X_{n}\right]\right)\left(X_{n+\ell }-E\left[X_{n}\right]\right),$$

where we set $N=10^{6}$.

**Figure 4:**
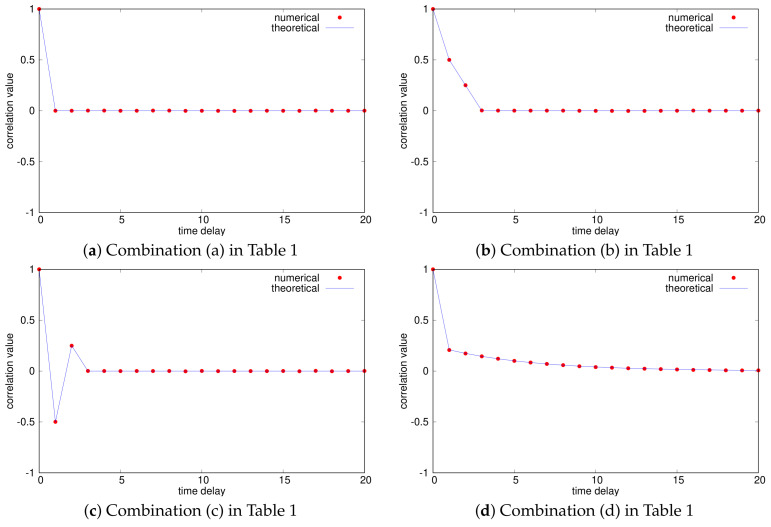

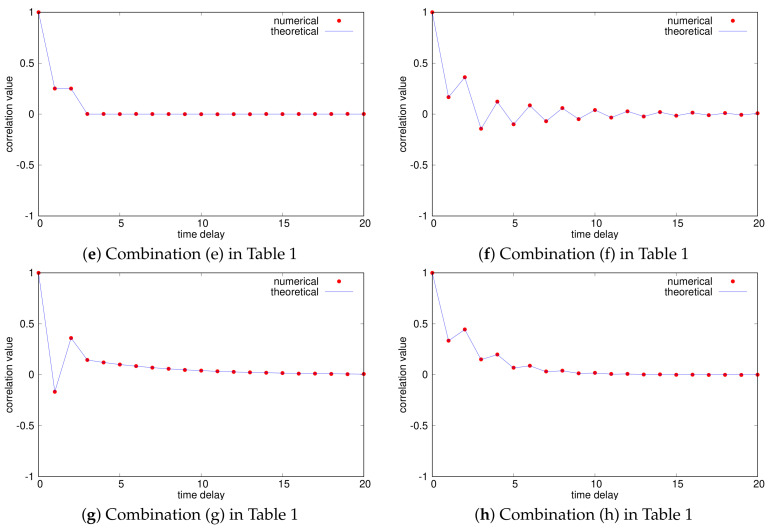
Normalized auto-correlation functions of *m*-bit random sequences (m=20).

**Figure 5:**
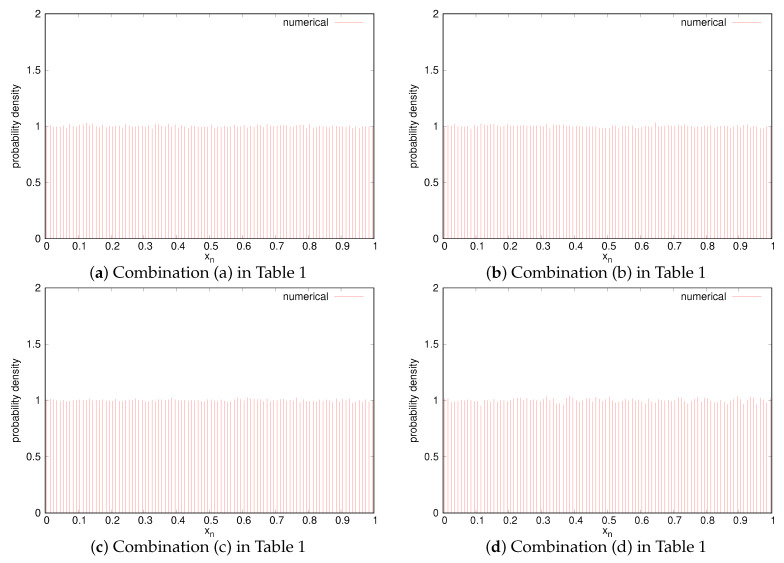

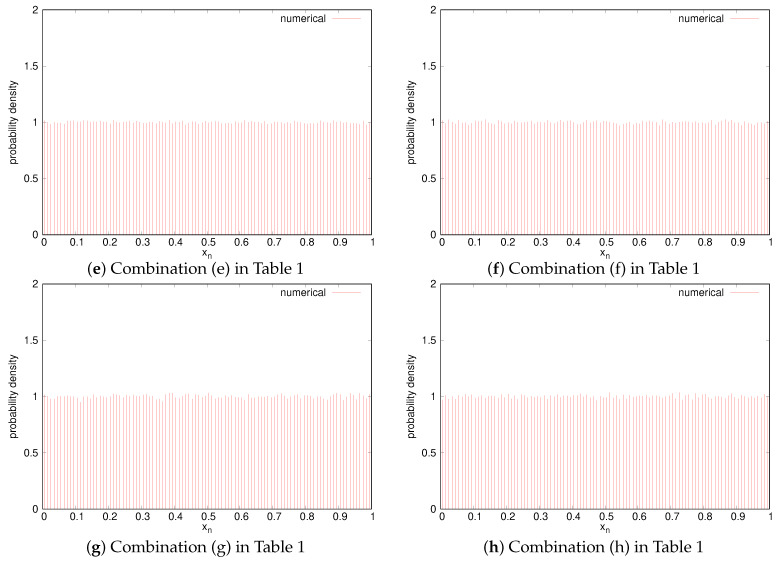
Probability densities of *m*-bit random sequences (m=20).

Figure 4 shows the numerical normalized auto-correlation functions $\hat{R}\left(\ell ;X_{n}\right)/\hat{R}\left(0;X_{n}\right)$, where m=20 and the theoretical auto-correlation functions obtained from (4) are also shown. We can confirm that the numerical auto-correlation values are almost equal to the corresponding theoretical values, which implies that the assumption of (5) is almost satisfied for our initial value selection. Additionally, it has been shown that we can generate *m*-bit sequences with a variety of auto-correlation functions by changing the chaotic binary sequences. Especially, the types of the auto-correlation properties of (d)–(h) cannot be realized using a simple chaotic map only. Furthermore, Figure 5 shows the probability distributions, where we used 100 bins [i/100,(i+1)/100) (i=0,1,⋯,99). We can find that the distributions are almost uniform, which implies that the assumption that the *m* binary sequences are independent of one another in Remark 3 is almost satisfied.

Next, we performed numerical experiments for the case m=3. In this case, we treated the sequences as $2^{3}$-ary integer sequences because *m* is small. Namely, we obtained a $2^{3}$-ary integer sequence as

(39) $${\hat{X}}_{n}=2^{m-1}B\left(x_{n}^{\left(1\right)}\right)+2^{m-2}B\left(x_{n}^{\left(2\right)}\right)+\cdots +2^{0}B\left(x_{n}^{\left(m\right)}\right).$$

**Figure 6:**
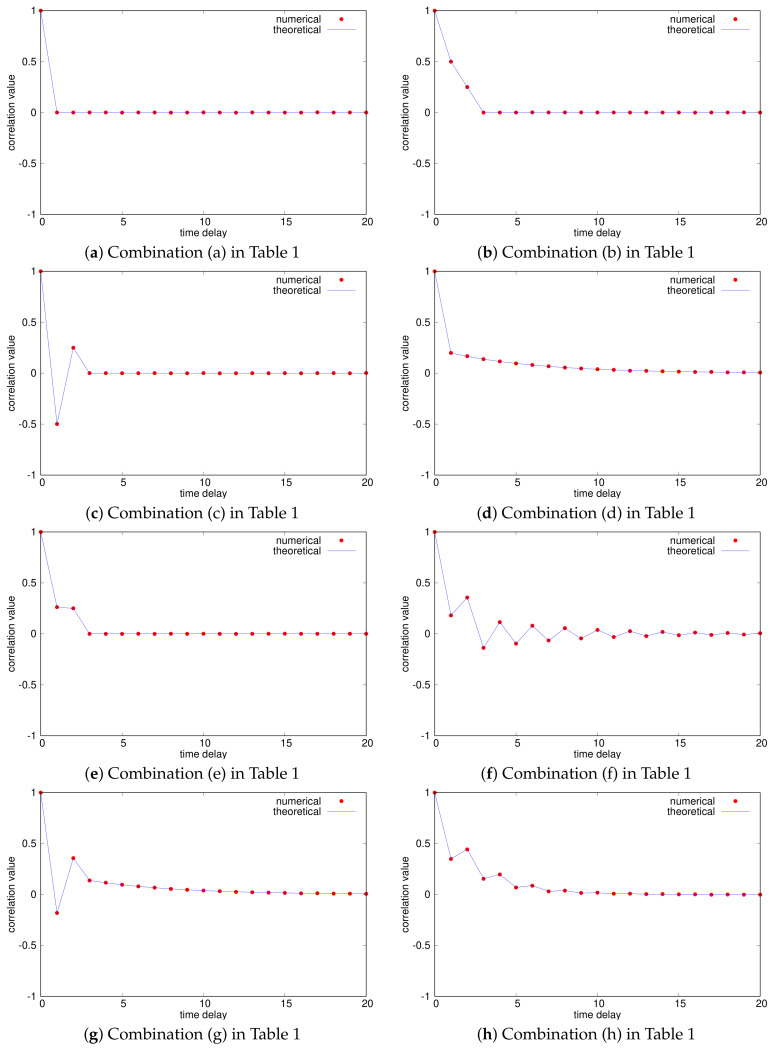
Normalized auto-correlation functions of *m*-bit random sequences (m=3).

We also generated eight kinds of $2^{3}$-ary sequences using eight combinations of chaotic binary sequences as in Table 1 and computed the numerical auto-correlation function $\hat{R}\left(\ell ;{\hat{X}}_{n}\right)$. Figure 6 shows the numerical normalized auto-correlation functions $\hat{R}\left(\ell ;{\hat{X}}_{n}\right)/\hat{R}\left(0;{\hat{X}}_{n}\right)$ of the $2^{3}$-ary sequences. We can find that they are almost equal to the auto-correlation functions for m=20. The details are slightly different due to the difference of the value of *m*. Table 2 shows the probability distributions, that is, the probabilities of the eight symbols (0∼7) in the $2^{3}$-ary sequences. The probability of each symbol is almost equal to $1/2^{3}$, which implies that the probability distribution is almost uniform.

## 5. Conclusions

We have discussed the auto-correlation functions of *m*-bit sequences obtained from *m* binary sequences. The theoretical auto-correlation function and the numerical functions show that we can easily generate *m*-bit (or $2^{m}$-ary) random numbers with a variety of auto-correlation properties from *m* chaotic binary sequences with prescribed auto-correlations. In our methods, the *m*-bit random numbers are obtained by controlling the auto-correlation of each bit sequence, which is quite unique and the main contribution of this paper. The application to Monte-Carlo methods and the discussion of higher-order statistics are left to future study.

## Figures and Tables

**Table 1:** Combinations for generating *m*-bit sequences.

	i=1	i=2∼m
(a)	ACF-1	ACF-1
(b)	ACF-2	ACF-2
(c)	ACF-3	ACF-3
(d)	ACF-1	ACF-4 (a=1.2)
(e)	ACF-2	ACF-3
(f)	ACF-2	ACF-4 (a=−1.2)
(g)	ACF-3	ACF-4 (a=1.2)
(h)	ACF-4 (a=1.5)	ACF-4 (a=−1.5)

**Table 2:** Probability distributions of each symbol (0∼7) in $2^{3}$-ary sequences (m=3).

Value	Probability							
($2^{3}$-ary Symbol)	(a)	(b)	(c)	(d)	(e)	(f)	(g)	(h)
0	0.125601	0.125200	0.125324	0.124573	0.125290	0.124844	0.124969	0.125310
1	0.125116	0.125883	0.124778	0.125444	0.124755	0.125496	0.125057	0.125829
2	0.124546	0.125090	0.124958	0.125049	0.124806	0.125352	0.125063	0.126000
3	0.125041	0.124091	0.125284	0.125238	0.125413	0.124572	0.125255	0.124875
4	0.124809	0.125149	0.124792	0.124936	0.124826	0.125353	0.124540	0.124821
5	0.124954	0.125055	0.124779	0.124460	0.124802	0.124458	0.124847	0.124540
6	0.125100	0.124851	0.124749	0.124727	0.124901	0.124467	0.124713	0.124196
7	0.124833	0.124681	0.125336	0.125573	0.125207	0.125458	0.125556	0.124429

## Data Availability

Data sharing is not applicable for this article.
